# Puerperal Fever

**Published:** 1873-09

**Authors:** J. E. O’Brien

**Affiliations:** Scranton, Pa.


ARTICLE IV.—*Puerperal Fever. Treatment by Intra-Uterine Disinfection.* By J. E. O'BRIEN, M.D., Scranton, Pa.

Having had several cases of puerperal fever during the past year, I have given them much study, and made an advance in treatment which I do not find on record; if the same should have escaped my limited researches, however, I beg to be directed to it.

In all the cases of puerperal fever, which I have seen, I have recognized the presence of a septic poison in the womb, the detritus of placenta, membranes, or clots; or pus, a result of inflammation caused by violence at the hands of the midwife. The axiom which I would give the profession, (if I have not been anticipated), is—

To neutralize the septic matter by *intra-uterine* injections of carbolic acid.

A very good illustration of the treatment usually employed, may be found in the JOURNAL, Vol. XXVI, p. 110, but nowhere have I found a suggestion of anything *beyond* opiates, eliminants, and disinfectant vaginal injections. The latter as recommended in our text books form a curious anomaly in practice, for it is not the vagina which absorbs the organic poison, but the open veins of the womb, or its absorbents now actively engaged in the process of involution.

The most serious objection to intra-uterine injections in gynæ-

cological practice, is the alarming colic which they produce unless their exit be provided for by previous dilatation with sponge tents; the syringe which has been constructed to return injected fluids is practically a failure. This objection, however, does not apply to the post-partem uterus, as it has been dilated, and is tolerant of fluids, and used to expelling them.

In a recent case of commencing puerperal fever, I injected into the womb four ounces of warm water, which washed out ropy, offensive pus; I then injected two ounces of warm water, containing half a dram of carbolic acid (crystals). The patient had a slight colic after the water injection, but none after the carbolic acid, and in a few minutes she said that the soreness of the womb, which had been very great, was easier; she then took carthartics, Dover's powder, had hot water, in flannel, to the abdomen, etc. The lochia, previously suppressed, occurred again immediately after the syringing, and continued profusely.

Six hours later the fever was less, the soreness of the abdomen much better, tongue cleaner, lochia continuing, and further injection, which I had determined upon, apparently not required. The next day she had stitches in the right and left hypochondrii, and one or two spells of faintness, but she made a good recovery. I would not attempt to prove anything by a single case, but I believe the proposition is self-evident that if we think there is septic matter in the womb we ought to reach it directly with disinfectants.

A syringe with a long tube, curved like a uterine sound, is necessary. (They are made with a return gutter, which, as I have said, is generally useless.)

